# MindTS-MMD: A Chinese Multimodal Dataset for Emotion Expression Analysis in Children with Tourette Syndrome

**DOI:** 10.1038/s41597-026-08006-4

**Published:** 2026-08-01

**Authors:** Jianping Wang, Haoyu Wu, Liping Li, Xiaoxia Fang, Qian Li

**Affiliations:** 1 School of Computer Science and Technology, Henan Institute of Science and Technology, Xinxiang, Henan 453003 China; 2 Department of Pediatrics, Xinxiang Central Hospital, Xinxiang, Henan 453000 China; 3 The Fourth Clinical College, Henan Medical University, Xinxiang, Henan 453000 China

## Abstract

Multimodal resources for emotion expression analysis in pediatric clinical populations remain limited, particularly for children with Tourette syndrome (TS). MindTS-MMD was developed as a Chinese multimodal dataset to support computational research on emotional expression and tic-related behavior in this population. The dataset contains 10,034 instance-level samples from 60 children with TS aged 6–12 years, collected through semi-structured emotion-elicitation tasks. Each sample includes available symbolic representations from three modalities—visual, acoustic, and semantic—and is linked to a corresponding annotation record. The annotations cover seven categories: anxious, calm, focused, irritable, relaxed, shy, and tense, together with child-reported and experimenter-observed valence–arousal ratings and tic occurrence, anatomical location, and frequency when observable. Annotation reliability, signal quality, audiovisual synchronization, facial tracking, modality completeness, and tic–emotion co-occurrence were evaluated. Unimodal and multimodal baselines are provided to illustrate the computational usability of the released representations.

## Background & Summary

Emotion expression is closely related to psychological well-being, social functioning, and behavioral adaptation, including in children with behavioral and developmental difficulties^1^. Its analysis is therefore important in affective computing, developmental psychology, and clinical mental health research. Facial behavior, speech, language, and physiological activity convey complementary aspects of emotion, and relying on a single modality may omit relevant information. Multimodal analysis combines information from these signal types for emotion recognition and expression modeling^2^.

Multimodal emotion datasets involving children remain limited. EmoReact^3^ demonstrated the feasibility of collecting audiovisual emotional responses from children. CALMED^4^ extended this work to children with autism through annotated audio and video data, while AKTIVES^5^ combined facial and physiological signals from children with different special needs for stress recognition. These datasets have supported pediatric affective computing, but existing resources do not adequately cover Chinese children with Tourette syndrome (TS) or provide aligned emotion and tic-behavior annotations for this population.

Datasets for adults and general populations are considerably more established. DEAP^6^ introduced a widely used benchmark combining electroencephalographic, peripheral physiological, and facial signals. SEWA-DB^7^ extended audiovisual affect analysis to more natural interaction settings. Large-scale facial resources such as WebFace260M^8^ have contributed to the development of facial representation methods, while the East Asian facial expression database^9^ highlighted the importance of population and cultural characteristics in expression recognition. EAV^10^ collected synchronized EEG, audio, and video data in conversational settings. MAHNOB-HCI^11^ combined physiological and audiovisual signals for affect recognition, and EmotionMeter^12^ provided a multimodal framework based on physiological and behavioral information.

Further physiological benchmarks include DREAMER^13^, MPED^14^, and ASCERTAIN^15^, which use EEG, ECG, galvanic skin response, and related signals for emotion analysis. AMIGOS^16^ extended affective research to individual and group settings. IEMOCAP^17^ captured multimodal emotion expression in dyadic interaction, while SEMAINE^18^ focused on emotionally colored conversations between people and virtual agents. NNIME^19^ provided a Chinese interactive multimodal emotion corpus. RAVDESS^20^, BAUM-1^21^, and SAVEE^22^ contributed acted, spontaneous, or semi-controlled audiovisual emotion samples. K-EmoCon^23^ collected multimodal signals during naturalistic debates, and PEGCONV^24^ examined emotion recognition in conversations using brain and physiological signals. Collectively, these datasets cover a range of adult and general-population settings, whereas multimodal resources for pediatric clinical populations remain less common.

MindTS-MMD^25^ was developed to address this gap. The dataset contains 10,034 instance-level records derived from 60 clinically diagnosed children with TS. It covers seven emotion-elicitation categories: anxious, calm, focused, irritable, relaxed, shy, and tense. Each instance contains available visual, acoustic, and semantic representations linked to a corresponding annotation record. The annotations include the emotion category, child-reported and experimenter-observed valence–arousal ratings, and tic occurrence, anatomical location, and frequency when observable. The released data comprise three symbolic channels—visual, acoustic, and semantic—derived from synchronized multimodal recordings.

MindTS-MMD^25^ supports subject-independent evaluation of unimodal and multimodal emotion classification, comparison of modality-specific contributions, and assessment of fusion strategies. Its aligned channels can be used to investigate multi-granularity cross-modal alignment^26^ and temporal affect modeling^27^. The emotion and tic annotations also allow analysis of emotion-related behavioral changes and transitions, complementing recent work on emotion-shift-aware dialogue modeling^28^. The participant-level organization of the dataset further permits evaluation of transfer-learning approaches^29^, few-shot emotion recognition^30^, and federated multimodal learning^31^. MindTS-MMD^25^ therefore provides a Chinese pediatric resource for studying multimodal emotional expression and its association with observable tic behavior in children with TS.

## Methods

MindTS-MMD^25^ was constructed through participant recruitment, semi-structured emotion-elicitation tasks, synchronized audiovisual acquisition, manual transcription and annotation, and extraction of instance-level symbolic multimodal representations. The procedures are described below.

### Participants

A total of 74 children with Tourette syndrome (TS) were recruited from collaborating hospitals between March and July 2024. Following the screening and feasibility assessment, 60 children met the eligibility criteria, completed the full experimental procedure, and were included in MindTS-MMD^25^. All diagnoses were confirmed by certified pediatric neurologists according to the criteria of the Diagnostic and Statistical Manual of Mental Disorders, Fifth Edition (DSM-5).

Eligible participants were 6–12 years of age, had a confirmed diagnosis of TS, and were able to understand and complete the semi-structured experimental tasks. Children with severe intellectual disability or other neurological conditions that could substantially interfere with emotional expression, language production, or task participation were excluded.

The included participants had a mean age of 9.24 ± 1.78 years and comprised 47 boys and 13 girls. The male-to-female ratio was approximately 3.6:1, which is consistent with the established male predominance in pediatric TS populations^32^. Additional demographic and clinical characteristics, including medication status, disease duration, and YGTSS total tic scores, are summarized in Table 1. These variables are provided only at the aggregate cohort level and are not included in the released instance-level records.

**Table 1:** Demographic and clinical characteristics of the included participants (N = 60).

Characteristic	Value
Age, years, mean ± SD	9.24 ± 1.78
Age range, years	6–12
Male, n (%)	47 (78.3%)
Female, n (%)	13 (21.7%)
Receiving tic-related medication, n (%)	18 (30.0%)
Disease duration since tic onset, months, median (IQR; range)	21 (14–30; 7–48)
YGTSS total tic score, mean ± SD	28.45 ± 5.33

### Study design and experiment protocol

This study employed a semi-structured interaction paradigm to collect speech, video, and text data from children with TS during controlled elicitation tasks. The tasks included viewing affective pictures, listening to and retelling stories, situational narration, role-playing, and responding to semi-structured interview prompts. The raw audiovisual recordings were used for annotation, quality control, and symbolic representation extraction and were not included in the released dataset.

The experimental protocol targeted seven categories for emotion-expression analysis: anxious, calm, focused, irritable, relaxed, shy, and tense. The selection of these categories was guided by experimental design considerations rather than by a single canonical taxonomy of basic emotions. Specifically, the categories were selected with reference to their intended valence–arousal characteristics and to reflect affective experiences commonly encountered in children’s daily social interactions^33^ and clinically relevant patterns reported in children with TS, including tension, anxiety, irritability, and social withdrawal^34^. This task-oriented and elicitation-driven scheme was applied consistently across all pediatric experimental sessions, with the intended valence-arousal characteristics and psychological attributes of each category provided in Table 2.

**Table 2:** Intended valence–arousal characteristics and psychological attributes of the seven emotion-elicitation categories.

Category	Intended valence	Intended arousal	Psychological attributes
Anxious	Negative	High	Worry and uneasiness, accompanied by physiological tension
Calm	Positive	Low	Emotional stability and relaxation
Focused	Positive	Medium	Sustained attention and task engagement
Irritable	Negative	High	Anger and agitation, prone to emotional outburst
Relaxed	Positive	Low	Pleasant and comfortable state, physiological relaxation
Shy	Negative	Medium	Withdrawal and timidity, accompanied by social anxiety
Tense	Negative	High	Stress-induced strain and heightened anxiety

The seven categories formed a task-oriented emotion-elicitation scheme. Most categories represented affective states, whereas focused was included to capture sustained attention and task engagement during structured activities and was therefore treated as a task-related state rather than a conventional discrete emotion. The labels refer to the intended emotional contexts of the tasks and the corresponding expressive responses assessed during annotation.

**Fig. 1:**
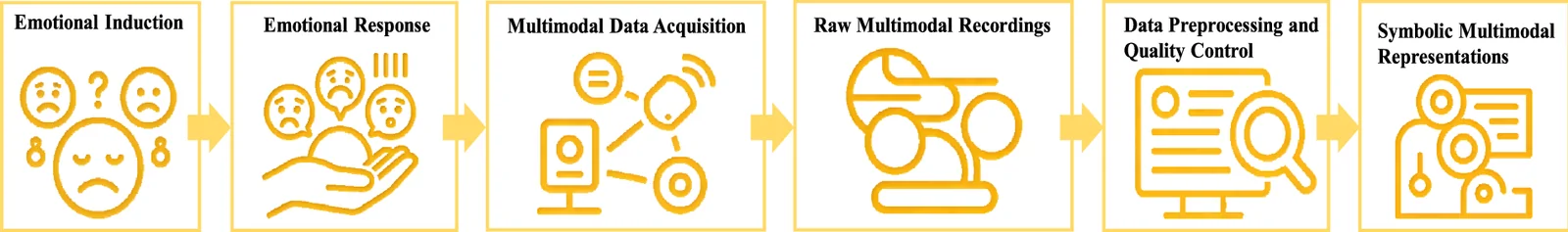
Workflow of multimodal data acquisition.

Each experimental task followed a common cycle of stimulus presentation, contextual guidance, and verbal or free expression. Children were first exposed to a selected stimulus and were then invited to respond through speech, facial expression, or gesture within a fixed time window. The overall workflow of multimodal data acquisition is shown in Fig. 1.

All candidate stimulus materials underwent small-scale pilot testing before the formal experiment and were individually reviewed by psychology specialists to assess their age appropriateness and consistency with the intended elicitation context. Task sequences were presented in a pseudo-randomized order to reduce carryover between successive conditions.

#### Session I: Screening session

Each child completed an individual screening session on the first day of participation. The session lasted approximately 50 min and was used to familiarize the child with the experimental procedure, identify suitable elicitation materials, and assess comprehension, emotional engagement, and comfort. The candidate material pool contained 70 task units, with 10 units prepared for each elicited state. Under the guidance of researchers and a clinical psychologist, each child selected three to five task units per state that produced the clearest observable response. Alternative materials were provided when the intended response was not observed.

Each child also completed a practice cycle of stimulus presentation and expression without data recording. Children who demonstrated adequate comprehension, engagement, and tolerance proceeded to the main experiment. The screening procedure is summarized in Table 3.

**Table 3:** Procedure of the screening phase.

Session I: Screening		
Procedure	Time (min.)	Description
Introduction	5	Researchers explained the study objectives, task formats, and possible psychological experiences to the child and guardian.
Material familiarization	10	Children were introduced to the pool of 70 candidate task units, with 10 units prepared for each elicited state.
Task selection	15–20	Children selected three to five task units per state under the guidance of researchers and a clinical psychologist. Alternative materials were provided when the intended response was not observed.
Practice trial	10	Children completed one practice cycle of stimulus presentation and expression without data recording.
Feasibility assessment	5	Researchers assessed comprehension, engagement, and comfort before determining whether the child could proceed to the main experiment.

#### Session II: Main experiment

**Fig. 2:**
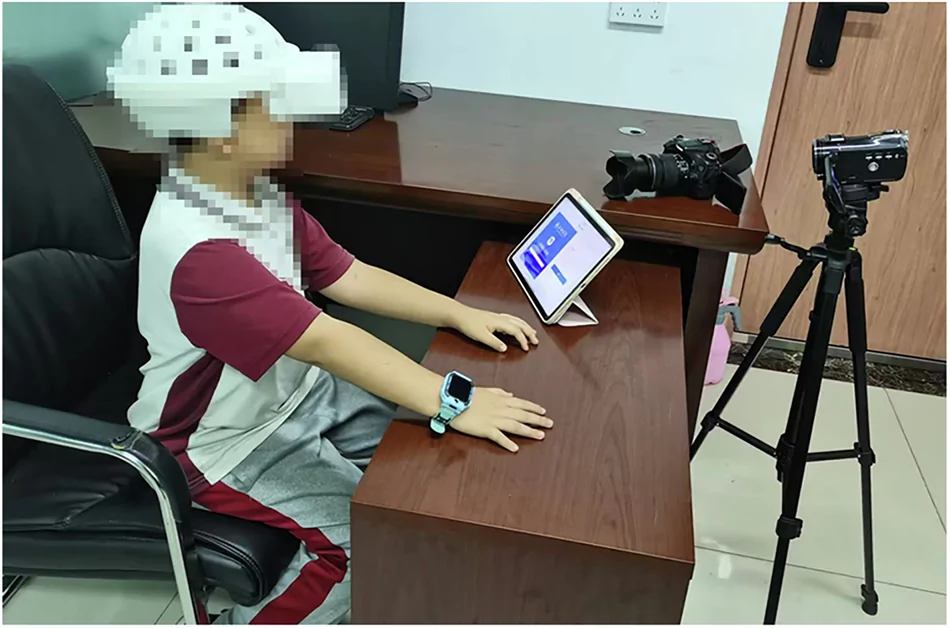
Experimental setup.

The main experiment was conducted individually on the day following the screening session to reduce fatigue and excessive familiarity with the selected materials. Each session lasted approximately 2 h and was conducted in a quiet, evenly lit laboratory. Only the researchers and a clinical psychologist were present during the session. The experimental setup, including the participant position and recording arrangement, is shown in Fig. 2.

Each participant completed 20 experimental iterations, with each iteration lasting approximately 4 min. The main experimental procedure is provided in Table 4. Across the complete session, each participant completed approximately 140–160 elicitation task units. Because a single task unit could produce more than one valid response segment during subsequent task-based segmentation, the number of task units was not identical to the number of released symbolic instances.

**Table 4:** Procedure of the main experimental phase.

Session II: Main Experiment		
Procedure	Time (min.)	Description
Experiment preparation	10	Researchers reviewed the task instructions, helped the child reach a stable baseline state, and checked the recording and lighting conditions.
Experimental iterations (20 rounds)	75–80	Each child completed 20 iterations. Each iteration contained one calm task and several additional elicitation tasks covering the target states. Following each stimulus, the child provided a verbal response or free expression within 20–30 s while audio and video were recorded synchronously.
Valence–arousal rating	15–20	After each iteration, the child completed a modified nine-point valence–arousal scale ranging from −4 to +4, while experimenters independently recorded observational ratings.
Tic annotation and behavioral observation	Concurrent	Tic behaviors and related behavioral information were recorded throughout the experiment.
Rest and adjustment	15–20	Rest periods were provided between iterations and could be adjusted according to the child’s condition.
Summary and feedback	10	Researchers collected feedback and confirmed that the child had returned to a stable emotional state.

Children were encouraged to respond using expressive speech, facial expressions, and gestures rather than reading the prompts directly. The sequence of tasks was pseudo-randomized, and calm tasks were used as transitional conditions to reduce residual effects from preceding high-arousal tasks.

**Fig. 3:**
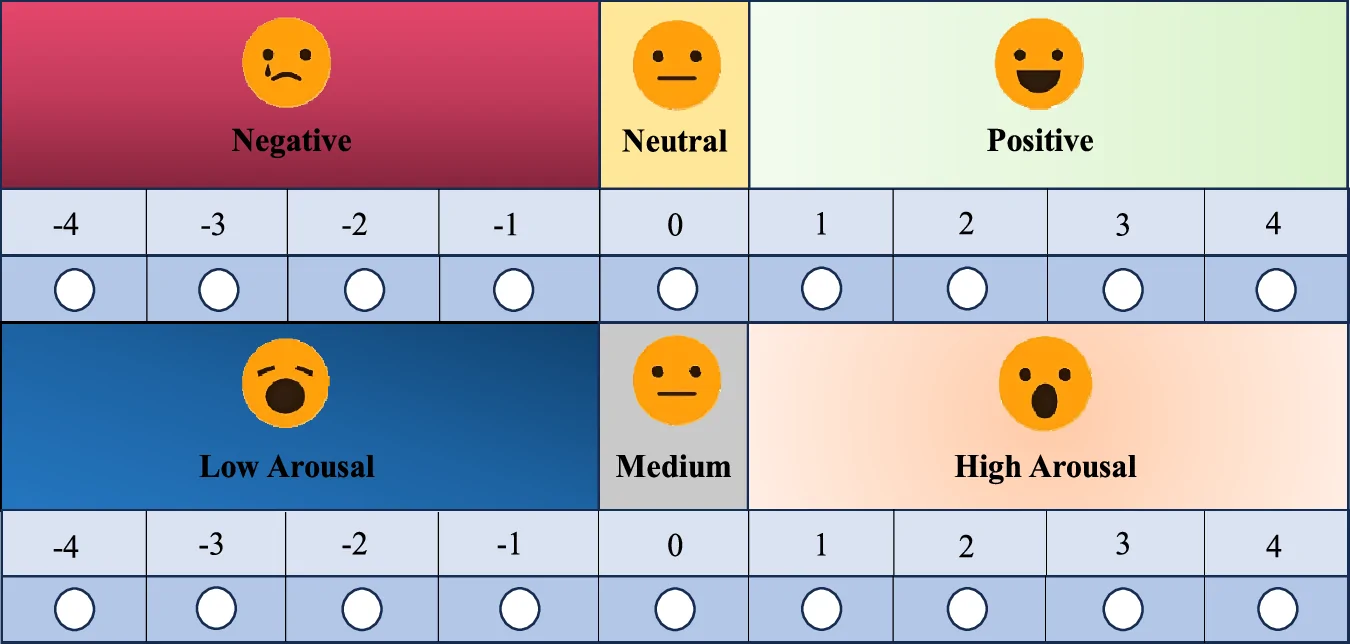
Valence–Arousal rating interface.

At the end of each iteration, participants rated their subjective valence and arousal using a modified nine-point scale ranging from −4 to +4. Facial illustrations and color cues were incorporated into the rating interface to help children understand the two affective dimensions. Experimenters provided standardized instructions and independently recorded observational valence and arousal ratings. The interface used for valence–arousal assessment is shown in Fig. 3.

Tic behaviors were observed throughout the experiment and subsequently documented in the instance-level annotations. Rest periods were provided between iterations, and the experiment was suspended if a child experienced discomfort or was unable to continue.

The responses in MindTS-MMD^25^ were elicited under a semi-structured experimental paradigm. They should therefore be interpreted as responses to predefined stimuli and interaction prompts rather than as fully spontaneous emotional expressions recorded in everyday settings.

##### Annotation procedure

Each released instance was independently reviewed by two trained annotators. During the initial review, the annotators were blinded to the intended elicited-state category and independently assessed the observable affective or task-related response. Their assessments were subsequently compared with the task-defined label, which served as the elicited-state label in the released annotation. Cases in which the observed response was considered inconsistent with or insufficiently representative of the intended state were subjected to further review.

Child valence and arousal ratings were obtained directly from the self-report scale during the experiment. Experimenter-observed valence and arousal ratings were independently recorded by two trained raters who were blinded to the child’s self-reported ratings. The original independent ratings were retained for the assessment of inter-rater reliability.

Tic annotations documented whether a tic occurred and, when observable, its anatomical location and frequency. These annotations were independently completed by two trained raters. Disagreements concerning elicited-state verification, experimenter-observed ratings, or tic annotations were first discussed by the relevant raters. Consensus decisions were adopted for the released annotations, and unresolved cases were adjudicated by a third domain expert. Inter-rater reliability was calculated from the original independent annotations before consensus resolution and is reported in the Technical Validation section.

### Data acquisition

All experimental sessions were conducted in a quiet, evenly illuminated laboratory under standardized recording conditions. Each participant was seated at a desk facing a tablet used to present the experimental materials. Two fixed high-definition digital cameras were positioned at frontal and front-oblique angles to capture facial expressions, head movements, gestures, and upper-body behavior. The camera positions and acquisition settings were kept consistent across sessions. Video was recorded at a resolution of 1920 × 1080 pixels and a frame rate of 25 frames per second, stored initially in AVI format, and subsequently transcoded to MP4 for internal processing and storage. Audio was recorded concurrently through the built-in high-sensitivity microphone of the primary recording camera at a sampling rate of 16 kHz and stored in lossless WAV format. The audiovisual recordings were aligned according to the experimental task timeline for subsequent segmentation and multimodal processing.

The semantic modality was generated through manual transcription of the recorded speech. Trained transcribers produced verbatim Chinese transcripts, which were independently cross-checked by two annotators against the corresponding audiovisual response segments. The verified transcripts were aligned with the relevant response intervals and stored as UTF-8-encoded TXT files during internal processing. Filled pauses and hesitation markers were retained within otherwise interpretable utterances. Accidental personally identifiable information, including personal names, locations, schools, hospitals, and other institutional references, was manually screened and removed or masked. Only de-identified transcripts were used for symbolic representation extraction and inclusion in the released dataset. The raw audiovisual recordings and intermediate transcription files were used for annotation, quality control, and symbolic representation extraction and are not included in the released MindTS-MMD^25^ archive.

### Symbolic representation extraction

The synchronized audiovisual recordings were converted into instance-level symbolic representations through task-based segmentation, modality-specific feature extraction, segment-level aggregation, and JSON packaging. Preparatory periods, researcher prompts, prolonged silence, and technically invalid intervals were removed before segmentation. Each retained segment corresponds to one valid response interval and is indexed by an anonymized participant identifier, iteration number, elicited-state label, and within-task segment number.

Visual representations were constructed using OpenFace 2.0 together with segment-level motion analysis. OpenFace outputs were obtained for each valid video frame and aggregated across the corresponding segment. The released visual channel contains 25 numerical descriptors covering seven facial action units, head-pose statistics, gaze behavior, eye-blink measures, global and regional motion, compact affect-related descriptors, and tracking-quality indicators. Frames with unsuccessful facial tracking were excluded from feature aggregation. Landmark success rate and mean tracking confidence were retained as quality indicators for each instance.

For the acoustic channel, all audio segments were resampled to 16 kHz before processing. A pretrained wav2vec 2.0 model, implemented using PyTorch 2.7.1, CUDA 11.8, and Hugging Face Transformers 4.55.4, was used during acoustic representation processing. In parallel, interpretable segment-level acoustic descriptors were extracted using custom Python scripts developed with Python 3.12.11, Librosa 0.11.0, NumPy 1.26.4, SciPy 1.16.2, and Praat-Parselmouth 0.4.7. The released acoustic representation comprises 18 numerical descriptors derived directly from each audio segment. These descriptors cover fundamental-frequency statistics, speaking rate, pause ratio, root-mean-square energy, voice-activity ratio, jitter, shimmer, harmonics-to-noise ratio, spectral centroid, spectral bandwidth, spectral roll-off, MFCC statistics, and acoustically derived valence- and arousal-related proxy measures. The proxy measures were computed solely from acoustic characteristics and were not generated from, fitted to, or otherwise informed by the manually annotated valence–arousal ratings.

The semantic channel contains the de-identified Chinese transcription aligned with each response segment. Pinyin and English versions are provided as auxiliary text to facilitate reuse. The keypoint summary field is a human-readable description derived from selected visual and acoustic descriptors. Because it incorporates information from multiple modalities, this field was treated as auxiliary documentation and excluded from all text-only and multimodal model inputs.

The released visual and acoustic channels contain 25 and 18 numerical descriptors, respectively, resulting in 43 numerical descriptors when both channels are available. Missing channels are explicitly marked in the missing-modality field and are not replaced with imputed values. Details of the extraction methods, feature categories, and dimensionality for the three modality channels are provided in Table 5.

**Table 5:** Composition of the released symbolic multimodal representations.

Channel	Extraction method	Released information	Dimension or format
Visual	OpenFace 2.0 and segment-level aggregation	Facial action units, head-pose statistics, gaze behavior, eye-blink measures, motion features, compact affect-related descriptors, and tracking-quality indicators	25 numerical descriptors
Acoustic	wav2vec 2.0 and custom Python acoustic-processing scripts	Prosody, energy, voice quality, spectral characteristics, MFCC statistics, and compact affect-related descriptors	18 numerical descriptors
Semantic	Manual transcription and de-identification	Chinese transcription, with pinyin and English versions as auxiliary text	Variable-length UTF-8 text

### Ethics approval and consent to participate

This study was conducted in accordance with the Declaration of Helsinki and relevant institutional ethical guidelines. The study protocol was reviewed and approved by the Medical Ethics Committee of Xinxiang Central Hospital (Approval No. 2022-114-01(K)). Written informed consent was obtained from the legal guardians of all participants for data collection and research use, as well as for the external sharing of de-identified, abstracted representations derived from the recordings for scientific research under a Data Usage Agreement. Age-appropriate assent was also obtained from each child before data collection.

The consent procedure explicitly distinguished between (i) the internal use of raw audiovisual recordings for annotation, quality control, and symbolic representation extraction and (ii) the external sharing of de-identified, task-oriented symbolic representations that do not expose raw visual or acoustic signals. All explicit personal identifiers, including names and contact information, were removed or masked before data release. No raw video frames, raw audio waveforms, or metadata enabling direct identity linkage are included in the released dataset.

## Data Records

The MindTS-MMD dataset^25^ has been deposited in Zenodo under the title “MindTS-MMD: A Chinese Multimodal Dataset for Emotion Expression Analysis in Children with Tourette Syndrome”. The dataset is available at https://doi.org/10.5281/zenodo.18281896.

### Repository organization

**Fig. 4:**
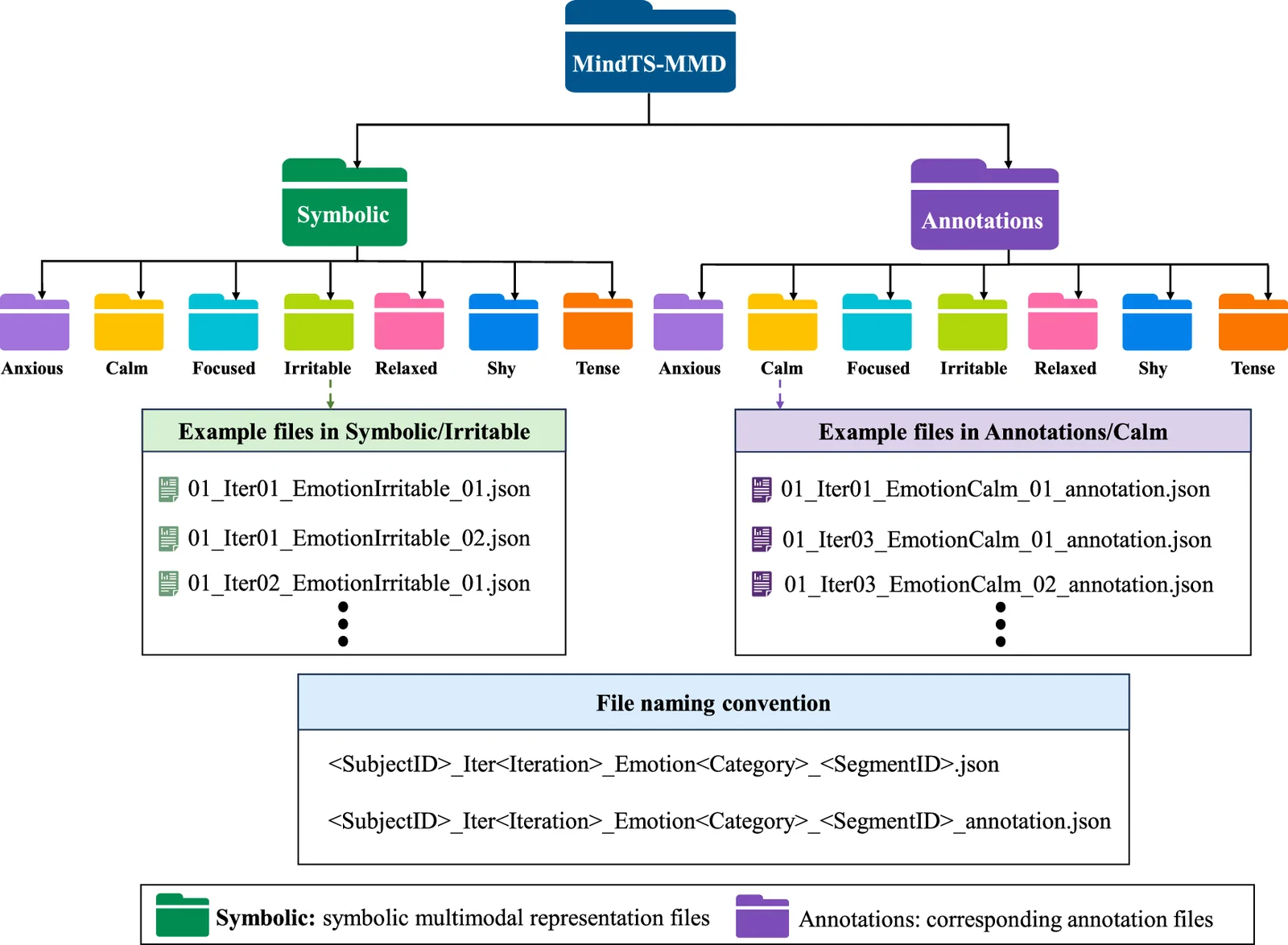
Repository organization and instance-level linkage between symbolic representation and annotation records in MindTS-MMD.

The archive contains two top-level directories, Symbolic and Annotations, as illustrated in Fig. 4. Each directory contains seven category-specific subdirectories: anxious, calm, focused, irritable, relaxed, shy, and tense. The Symbolic directory stores the released multimodal representation files, whereas the Annotations directory stores the corresponding affective and behavioral annotation files. Each symbolic file is paired one-to-one with an annotation file through the same instance identifier embedded in the filename. Symbolic files follow the naming convention:

<SubjectID>_Iter<Iteration>_Emotion<Category>_<SegmentID>.json

Annotation files follow the naming convention:

<SubjectID>_Iter<Iteration>_Emotion<Category>_<SegmentID>_annotation.json

where SubjectID identifies the anonymized participant, Iteration records the experimental iteration, Category specifies the target category, and SegmentID indexes the response segment within the corresponding elicitation task. The symbolic and annotation files share the same SubjectID, Iteration, Category, and SegmentID values, enabling one-to-one matching between paired records. SegmentID differentiates multiple valid response segments obtained from the same elicitation task. Participant identifiers were assigned at random and contain no personal, demographic, or clinical information.

### JSON structure and metadata

All released files are stored as UTF-8-encoded JSON objects. Each symbolic representation file contains the top-level keys subject_id, iteration, emotion, segment_index_num, visual_features, acoustic_features, text_content, and missing_modalities. The visual_features and acoustic_features objects are further organized into modality-specific descriptor groups, while text_content stores the de-identified Chinese transcript, pinyin, English translation, and auxiliary keypoint summary. The corresponding annotation file stores participant and iteration metadata, the emotion category, child-reported and experimenter-observed valence–arousal ratings, tic occurrence, anatomical location and frequency label, relative source-record paths, and modality-availability information. A null value in the missing-modality field indicates that all expected channels are available.

### Record contents

Each symbolic record corresponds to one response segment and includes the anonymized participant identifier, experimental iteration, elicited-state category, segment index, and available visual, acoustic, and semantic representations.

The semantic representation contains the de-identified utterance aligned with the response segment. For example, one released record contains the pinyin transcription “yǒu shíhou wǒ xiǎng fā huǒ, shēngyīn chǎochǎo nào nào de shíhou, wǒ xiǎng bǎ shū hé shàng,” translated as “Sometimes I feel like getting angry; when the environment becomes noisy, I want to close the book.” Filled pauses and hesitation markers, such as “en” and “wu,” are retained within interpretable utterances to preserve characteristics of the children’s natural speech. When a segment consists solely of non-lexical vocalizations without interpretable linguistic content, the semantic representation is marked as unavailable.

Each annotation record is linked to its symbolic record through the shared instance identifier. It contains the emotion label, child self-reported and experimenter-observed valence and arousal ratings, tic-behavior annotations, relative source-record references, and modality-availability metadata. Tic annotations indicate whether a tic is observed and, when available, specify its anatomical location and frequency. The relative source-record references are retained for internal traceability, while the corresponding raw audio and video recordings are not included in the released archive.

Modality availability is documented separately for each instance. A null value indicates that all expected channels are present. When a channel is unavailable because of recording interruption, non-response, technical failure, or insufficient valid content, the affected modality is explicitly identified, while the remaining data for that instance are retained. Missing channels are not replaced with synthetic or imputed values. The principal contents and data formats of the symbolic and annotation records are provided in Table 6.

**Table 6:** Content and format of the released JSON records.

Record type	Principal contents	Data format
Symbolic representation	Instance identifiers and available visual, acoustic, and semantic representations, together with modality-availability information	Numerical values, categorical entries, and UTF-8 text
Annotation	Emotion label, child- and experimenter-rated valence and arousal, tic occurrence, anatomical location and frequency, source-record references, and modality-availability information	Boolean, categorical, numerical, and text entries

### Data Overview

MindTS-MMD^25^ comprises 10,034 instance-level symbolic representation records and 10,034 corresponding annotation records derived from 60 children with Tourette syndrome. The dataset covers seven emotion categories—anxious, calm, focused, irritable, relaxed, shy, and tense—and three released modality channels: visual, acoustic, and semantic. Each symbolic record is linked one-to-one with its corresponding annotation record through a shared instance identifier. The complete archive has a total size of approximately 36.3 MB. The number of instances per emotion category ranges from 1,428 to 1,443. Channel-specific availability and missingness are reported in the Technical Validation section.

### File access and parsing

The released records follow standard JSON syntax and can be inspected using any JSON-compatible software. No proprietary software is required. In Python, the files can be loaded using the standard json library and paired through their shared instance identifiers. Example scripts for reading the records, matching symbolic representations with their corresponding annotations, and preparing the data for downstream analyses are provided in the accompanying code repository.

## Technical Validation

Technical validation focused on the reliability of the annotations, the quality and alignment of the source recordings, the completeness of the released modality channels, and the computational usability of the symbolic multimodal representations. Inter-rater agreement, audio signal-to-noise ratio, audiovisual synchronization error, facial-tracking success, modality availability, and tic-related descriptive statistics were examined as data-quality indicators. Baseline classification experiments were additionally conducted using the released JSON-based symbolic representations and de-identified text to demonstrate their suitability for computational analysis rather than to establish state-of-the-art performance.

### Annotation reliability

Inter-rater reliability was calculated from the original independent assessments completed by two trained annotators before consensus resolution. For the seven elicited affective and task-related states—anxious, calm, focused, irritable, relaxed, shy, and tense—the overall Cohen’s κ was 0.78. Category-specific κ values ranged from 0.71 to 0.80, with values of 0.76 for anxious, 0.80 for calm, 0.78 for focused, 0.73 for irritable, 0.72 for relaxed, 0.71 for shy, and 0.75 for tense.

Inter-rater reliability for the experimenter-observed valence and arousal ratings was assessed using a two-way random-effects, single-measure intraclass correlation coefficient. The ICC (2,1) values were 0.74 for valence and 0.71 for arousal. Children’s self-reported valence and arousal ratings were not included in this analysis because they reflected each participant’s subjective experience rather than repeated observer ratings of the same construct. The self-reported and experimenter-observed ratings were therefore retained as separate annotation fields.

### Signal and alignment quality

Audio quality was evaluated at the segment level using the signal-to-noise ratio (SNR). Speech-active and non-speech intervals were identified using voice-activity detection. For the i-th audio segment, the SNR was calculated as (1):

1 $$\mathrm{SN}{\mathrm{R}}_{i}=10{{\log }}_{10}\left(\frac{\frac{1}{{N}_{i}^{s}}{\sum }_{n=1}^{{N}_{i}^{s}}{\left({x}_{i,n}^{s}\right)}^{2}}{\frac{1}{{N}_{i}^{n}}{\sum }_{n=1}^{{N}_{i}^{n}}{\left({x}_{i,n}^{n}\right)}^{2}}\right)$$

where ${x}_{i,n}^{s}$ and ${x}_{i,n}^{n}$ are the n-th audio samples within the speech-active and non-speech intervals of the i-th segment, respectively, and ${N}_{i}^{s}$ and ${N}_{i}^{n}$ denote the corresponding numbers of samples. The segment-level SNR values were summarized using the median and interquartile range. Across the evaluated audio segments, the median SNR was 12.4 dB, with an interquartile range of 9.1–15.6 dB.

Audiovisual synchronization was evaluated by comparing corresponding speech-onset landmarks in the audio and video streams. For each evaluated segment, the onset of audible phonation and the onset of the corresponding visible lip movement were identified on the shared recording timeline. The absolute synchronization error of the i-th segment was given by (2):

2 $${e}_{i}=\left|{t}_{i}^{a}-{t}_{i}^{v}\right|$$

where ${t}_{i}^{a}$ and ${t}_{i}^{v}$ denote the audio-based and video-based speech-onset timestamps, respectively. The absolute synchronization errors were summarized using the mean and standard deviation, yielding an average temporal offset of 23 ± 9 ms.

Visual tracking quality was evaluated using the frame-level tracking status generated by OpenFace 2.0. The facial-landmark tracking success rate was defined as the proportion of successfully tracked frames among all evaluated frames. Facial landmarks were successfully tracked in 97.6% of the evaluated video frames.

### Modality completeness

**Fig. 5:**
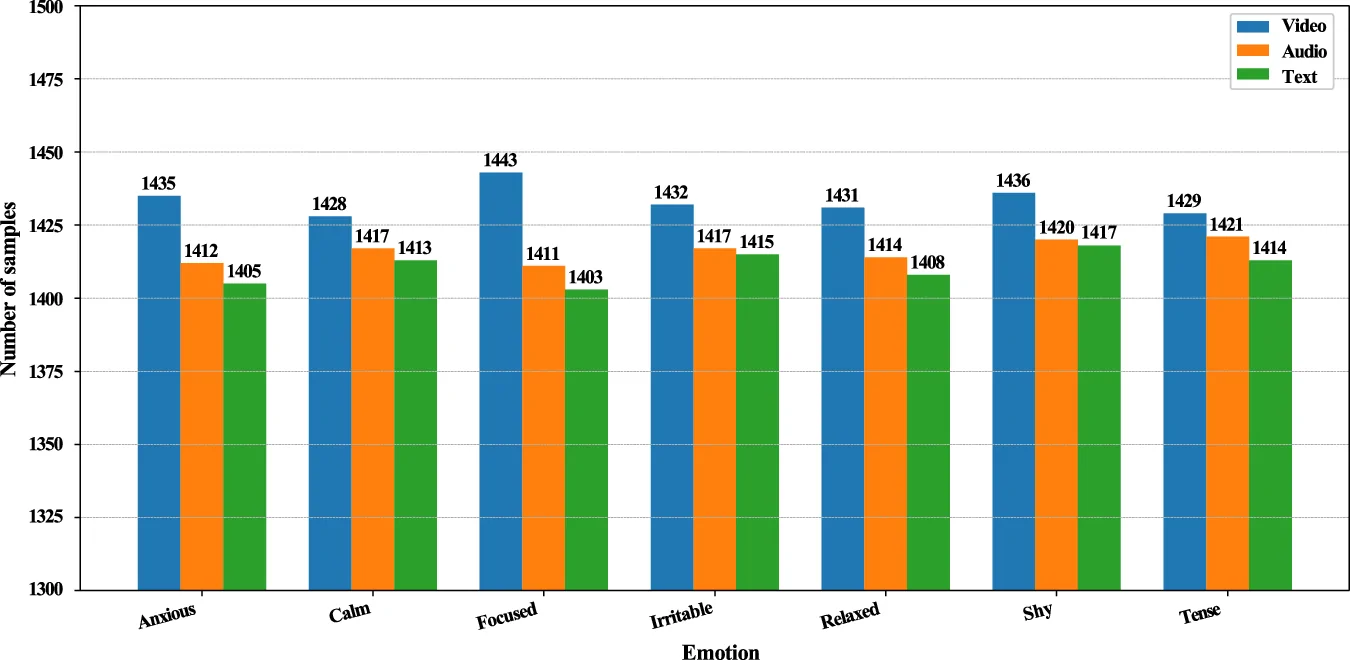
Distribution of available instances across the seven elicited states and released modality channels.

Modality completeness was assessed across the 10,034 released symbolic instances. At the category level, the number of available instances ranged from 1,428 to 1,443 for the visual channel, from 1,411 to 1,421 for the acoustic channel, and from 1,403 to 1,417 for the semantic channel, as shown in Fig. 5.

The overall availability and missingness of each modality are provided in Table 7. Missing acoustic or semantic channels mainly resulted from recording interruptions, participant non-response, technical issues during individual tasks, or the absence of interpretable linguistic content. Instances with partial modality availability were retained when the remaining channels contained valid information. Missing channels were explicitly indicated in the corresponding annotation records and were not imputed, while the available channels remained aligned with the same task-based response segment.

**Table 7:** Availability and missingness of the released modality channels in MindTS-MMD.

Released channel	Available instances	Missing instances	Missing rate (%)
Visual	10,034	0	0.00
Acoustic	9,912	122	1.22
Semantic (Text)	9,875	159	1.58

### Tic-related descriptive validation

Tic-emotion co-occurrence was examined using the instance-level tic annotations. A tic-positive instance was defined as a response segment in which at least one observable tic behavior was recorded. All 10,034 released instances contained a valid tic-occurrence annotation, and tic behaviors were observed in 3,271 instances, corresponding to an overall occurrence rate of 32.60%.

Descriptively, tic occurrence was not evenly distributed across the seven emotion categories. Higher occurrence rates were observed in high-arousal or clinically relevant categories such as tense and irritable, whereas lower rates were observed in calm and relaxed states. These findings provide a descriptive overview of observable tic-behavior distribution across the emotion categories represented in MindTS-MMD25, with category-level counts and occurrence rates provided in Table 8.

**Table 8:** Co-occurrence of tic behaviors across the seven emotion categories.

Category	Total instances	Tic-positive instances	Tic-negative instances	Tic occurrence rate (%)
Anxious	1,435	509	926	35.47
Calm	1,428	354	1,074	24.79
Focused	1,443	441	1,002	30.56
Irritable	1,432	589	843	41.13
Relaxed	1,431	295	1,136	20.61
Shy	1,436	482	954	33.57
Tense	1,429	601	828	42.06
Total	10,034	3,271	6,763	32.60

### Baseline utility assessment

Baseline experiments were conducted to assess the computational usability of the released symbolic multimodal representations rather than to pursue state-of-the-art performance. All experiments used only the released visual and acoustic representations and the de-identified Chinese transcriptions. In this section, the transcriptions contained in the released semantic channel are referred to as the text modality because they were directly processed as textual model inputs. The prediction task involved seven-class emotion classification.

The dataset was divided into training, validation, and test sets at a subject-independent ratio of 8:1:1, corresponding to 48, 6, and 6 participants, respectively. All instances from the same participant were assigned to a single subset, and the same split was used for all models. Numerical feature normalization was performed using statistics estimated from the training set. Each experiment was repeated using five random seeds—42, 777, 1234, 2025, and 3407—and the results are reported as mean ± standard deviation.

Four unimodal baselines were evaluated across the three released modality channels. Visual and acoustic feature dictionaries were flattened into fixed-length vectors using a deterministic feature order and separately classified using lightweight multilayer perceptrons with two hidden layers, ReLU activation, and dropout. Each symbolic instance corresponded to a complete task-based response segment and was directly assigned one prediction without frame-level aggregation or temporal voting. For the text modality, a character-level BiGRU was used as the lightweight baseline, while Chinese BERT was included as a pretrained comparison to further evaluate the semantic information retained in the released Chinese transcriptions.

For multimodal modeling, visual and acoustic representations were encoded using feedforward networks, while the text input was encoded using the BiGRU. The modality-specific representations were concatenated and passed to a shallow fully connected classifier. Binary modality masks were applied when acoustic or text channels were unavailable. Chinese BERT was evaluated only as a unimodal text baseline and was not used in the multimodal fusion experiments.

All models were trained using cross-entropy loss and the AdamW optimizer, with a batch size of 8 and a maximum of 200 epochs. Early stopping was applied when validation accuracy did not improve for 10 consecutive epochs, and the weights associated with the highest validation accuracy were retained. Model-specific learning rates and weight-decay settings were used. Accuracy, Macro-F1, and Cohen’s κ were reported as evaluation metrics.

The unimodal baseline results indicated that the visual MLP performed best among the non-pretrained models, as shown in Table 9. Chinese BERT further achieved an accuracy of 73.9% ± 3.8, a Macro-F1 of 0.71 ± 0.04, and a Cohen’s κ of 0.68 ± 0.03, exceeding the character-level BiGRU on three evaluation metrics.

**Table 9:** Performance of the unimodal baselines on emotion classification.

Input channel	Model	Accuracy (%)	Macro-F1	Cohen’s κ
Visual	MLP	64.3 ± 4.8	0.62 ± 0.06	0.49 ± 0.05
Acoustic	MLP	57.8 ± 4.2	0.56 ± 0.05	0.42 ± 0.04
Text	BiGRU	55.1 ± 3.9	0.54 ± 0.04	0.39 ± 0.04
Text	Chinese BERT (pretrained)	73.9 ± 3.8	0.71 ± 0.04	0.68 ± 0.03

**Fig. 6:**
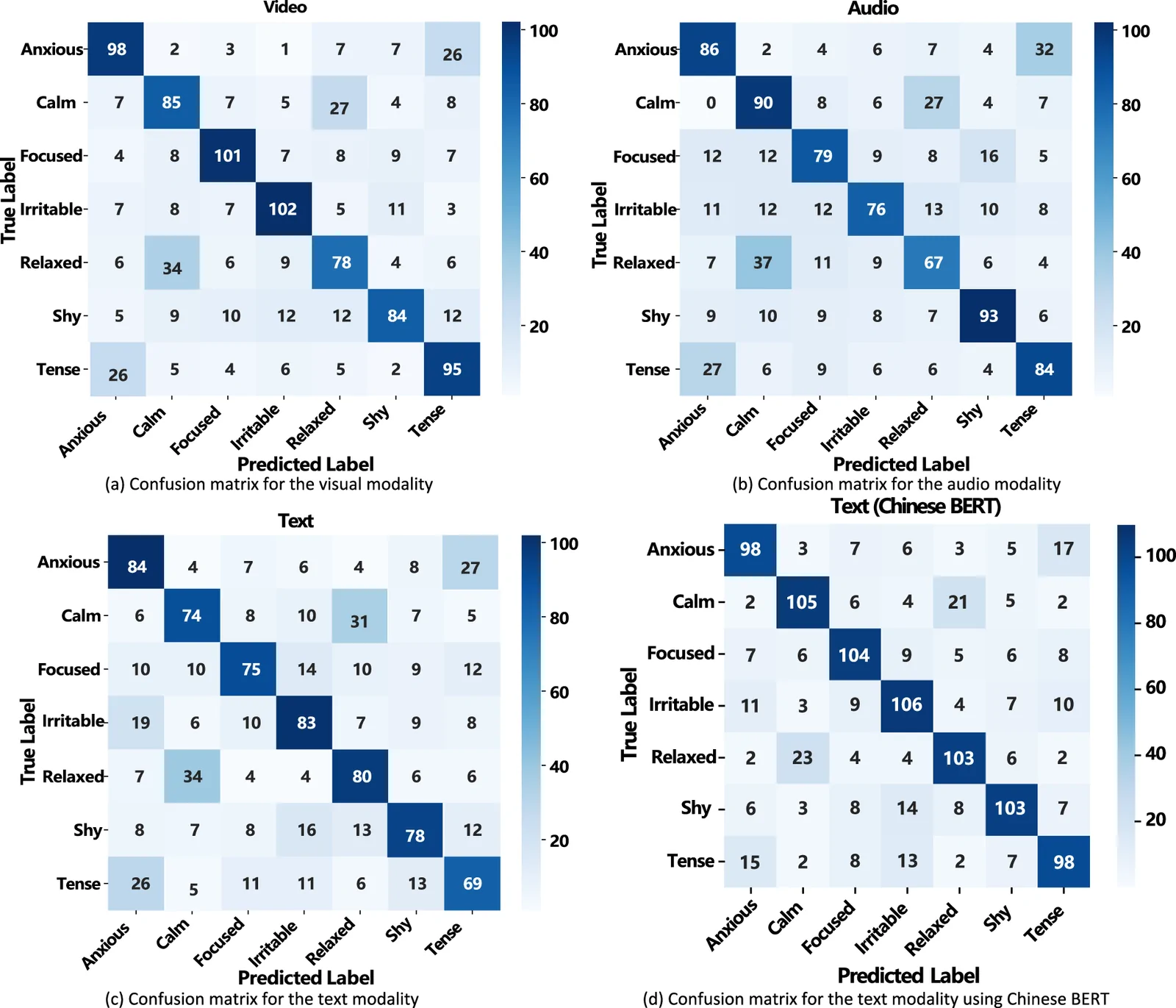
Confusion matrices of the four single-modality baseline models on the test set for emotion classification.

The four unimodal baseline models showed distinct category-level classification patterns on the test set. The most frequent misclassifications were observed between anxious and tense, and between calm and relaxed, as shown in Fig. 6. These category pairs are located in overlapping regions of the valence–arousal space and may share similar expressive characteristics under elicited conditions, making them difficult to distinguish using a single modality^35^. For Chinese BERT, the confusion matrix further reflects the extent to which category-discriminative semantic information was preserved in the Chinese transcriptions and provides a comparison with the character-level BiGRU under a pretrained language-modelling framework.

The multimodal fusion experiments indicate that combining acoustic and visual features improved performance compared with the corresponding unimodal MLP baselines, as shown in Table 10. The tri-modal configuration achieved the highest scores among all evaluated fusion settings, suggesting the complementary value of the released visual, acoustic, and text channels for joint modeling.

**Table 10:** Performance of the multimodal fusion baselines on emotion classification.

Input combination	Accuracy (%)	Macro-F1	Cohen’s κ
A + V	69.2 ± 4.5	0.67 ± 0.05	0.55 ± 0.05
A + T	61.0 ± 4.0	0.59 ± 0.04	0.46 ± 0.04
V + T	66.1 ± 4.3	0.64 ± 0.05	0.51 ± 0.05
A + V + T	71.8 ± 4.6	0.69 ± 0.05	0.58 ± 0.05

A, V, and T denote the acoustic, visual, and text inputs, respectively. The text input was derived from the released semantic channel and encoded using the BiGRU in all multimodal fusion baselines.

**Fig. 7:**
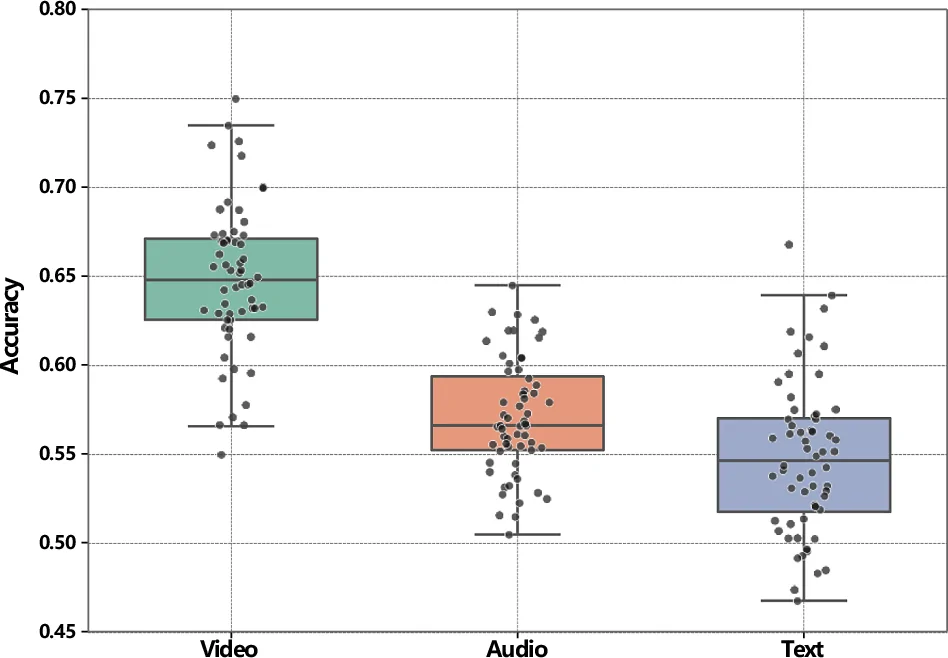
Participant-level accuracy distributions for each modality based on subject-wise out-of-fold predictions (n = 60).

Participant-level classification accuracy was further calculated for all 60 participants using the out-of-fold predictions from the auxiliary subject-wise evaluation, with one accuracy value obtained for each participant. These distributions provide a descriptive view of inter-individual variability in classification performance across the evaluated unimodal baselines, as shown in Fig. 7.

## Usage Notes

MindTS-MMD^25^ can be used for unimodal or multimodal analysis of emotion expression. The acoustic channel is suitable for speech-based modeling, the visual channel for facial and behavioral analysis, and the semantic channel for text-based classification. Symbolic records and annotation records can be matched through their shared instance identifiers. Scripts for loading, pairing, preprocessing, and baseline modeling are provided in the accompanying code repository.

The semantic channel contains de-identified Chinese transcriptions, with pinyin and English versions supplied as auxiliary text. Researchers may select the representation that best fits their task. The keypoint summary field should not be used as input to text-only models because it is derived from selected visual and acoustic descriptors and may introduce cross-modal information. Modality availability is recorded for each instance. When an acoustic or semantic channel is unavailable, the affected modality is identified explicitly and the remaining valid channels are retained. Missing entries should not be interpreted as zero-valued observations.

Subject-independent partitioning is recommended to avoid information leakage across children. All records from the same participant should remain within a single training, validation, or test subset. The baseline experiments reported here are intended as reproducible reference implementations and do not prescribe a particular modeling strategy.

Alongside the seven emotion categories, the annotations include child-reported and experimenter-observed valence–arousal ratings, as well as tic occurrence, anatomical location, and frequency when observable. These fields enable categorical emotion recognition, dimensional affect analysis, and investigation of associations between emotional expression and tic behavior. The dataset is intended for research and methodological development and should not be used for diagnosis or as a substitute for clinical assessment. All use must comply with the Data Usage Agreement and applicable ethical, legal, and institutional requirements. Participant re-identification, cross-dataset identity linkage, inference of personal identity attributes, and unauthorized redistribution are prohibited.

### Limitations

Demographic and clinical characteristics, including medication status, disease duration, and YGTSS total tic scores, are available only as aggregate cohort statistics and are not included in the released instance-level records. The current release therefore does not support participant-level stratification by tic severity, medication status, disease duration, or related clinical subtypes. This restriction was adopted to reduce the risk of indirect identification in a pediatric clinical cohort.

The data were collected through semi-structured tasks under controlled elicitation conditions. This design improved procedural consistency across participants, but the resulting expressions may be less spontaneous than those observed in everyday interactions^36^ and may not fully reflect behavior in natural social settings^37^. Findings obtained from MindTS-MMD^25^ should therefore be validated carefully before being transferred to real-world applications.

The cohort was limited to clinically diagnosed children with TS between 6 and 12 years of age. Although this provides a focused pediatric sample, it also limits generalization to adolescents, adults, children without TS, and populations with different demographic or cultural characteristics^38^. Models trained on this dataset may consequently show reduced performance when applied across populations^39^.

The released data consist of de-identified, abstracted symbolic representations rather than raw audiovisual recordings. This release design avoids the distribution of raw facial images and voice recordings but removes part of the temporal and expressive detail present in the original signals. Visual descriptors may not preserve all fine-grained facial and motion dynamics, particularly when tic-related movements are present. Acoustic descriptors may omit subtle temporal or prosodic cues, while the semantic channel varies in length and richness because of individual differences in children’s language production. These factors may contribute to modality-specific performance variation and complicate multimodal fusion^40,41^.

Child-reported and experimenter-observed valence–arousal ratings reflect different perspectives. Self-reports capture subjective experience, whereas observer ratings are based primarily on visible behavior. Disagreement between the two may affect analyses that assume direct correspondence, although their joint availability also allows subjective and observable affective responses to be compared^42^. The baseline experiments were designed to provide transparent reference values rather than exhaustively optimize performance. They include lightweight visual and acoustic multilayer perceptrons, a character-level BiGRU, a pretrained Chinese BERT baseline, and shallow multimodal fusion models. More advanced pretraining, temporal modeling, augmentation, missing-modality learning, and optimization strategies were not systematically examined.

MindTS-MMD^25^ is a controlled-access dataset comprising de-identified symbolic representations and aligned affective and tic-behavior annotations from Chinese children with TS. Future work may incorporate participant-level clinical measures under appropriate governance, collect data in more naturalistic settings^43,44^, broaden age and cultural coverage^45^, include a wider range of emotional expressions^46^, and investigate more advanced multimodal and personalized modeling approaches^47^.

## Data Availability

The MindTS-MMD dataset is hosted in the Zenodo repository under the title “MindTS-MMD: A Chinese Multimodal Dataset for Emotion Expression Analysis in Children with Tourette Syndrome”^25^ and is available through controlled access at https://doi.org/10.5281/zenodo.18281896. The released archive is approximately 36.3 MB and contains two top-level directories, Symbolic and Annotations, comprising UTF-8-encoded JSON files. The files can be parsed using standard JSON-compatible software or the Python standard-library json module without proprietary dependencies. Researchers requesting access are required to complete the Data Usage Agreement form at https://forms.gle/tZ6ERCxZzh6wPAbB9, provide their full name, institutional affiliation, and position or title, and agree to the stated conditions of use. Access may be granted to bona fide users for legitimate research purposes. Users remain responsible for complying with the ethical, legal, and institutional requirements applicable to their own research. The released dataset contains only de-identified symbolic multimodal representations and annotations. Raw audio and video recordings are not available to external users, including under controlled access. The Data Usage Agreement prohibits participant re-identification, cross-dataset identity linkage, inference of personal identity attributes, and unauthorized redistribution of the data.

## Code availability

The code associated with this study is publicly available in the MindTS-MMD GitHub repository (https://github.com/wangjianpinghist/MindTS-MMD-CODE). The repository provides complete implementations of the baseline experiments, including modeling code for the three individual modalities (visual, acoustic, and semantic), as well as multimodal experiments integrating all three modalities.
